# Differential cytotoxicity of long-chain bases for human oral gingival epithelial keratinocytes, oral fibroblasts, and dendritic cells

**DOI:** 10.1016/j.dib.2015.08.025

**Published:** 2015-09-07

**Authors:** Leslie A. Mehalick, Christopher Poulsen, Carol L. Fischer, Emily A. Lanzel, Amber M. Bates, Katherine S. Walters, Joseph E. Cavanaugh, Janet M. Guthmiller, Georgia K. Johnson, Philip W. Wertz, Kim A. Brogden

**Affiliations:** a Department of Periodontics, College of Dentistry, The University of Iowa, Iowa City, IA 52242, USA; b Dows Institute for Dental Research, College of Dentistry, The University of Iowa, Iowa City, IA 52242, USA; c Department of Oral Pathology, Radiology and Medicine, College of Dentistry, The University of Iowa, Iowa City, IA 52242, USA; d Central Microscopy Research Facility, The University of Iowa, Iowa City, IA 52242, USA; e Department of Biostatistics, College of Public Health, The University of Iowa, Iowa City, IA 52242, USA; f College of Dentistry, University of Nebraska Medical Center, Lincoln, NE 68583, USA

**Keywords:** Cytotoxicity, Human oral gingival epithelial keratinocytes, Oral gingival fibroblasts, Dendritic cells, Oral squamous cell carcinoma cells, Sphingosine, Dihydrosphingosine, Phytosphingosine, Glycerol monolaurate

## Abstract

Long-chain bases, found in the oral cavity, have potent antimicrobial activity against oral pathogens. In an article associated with this dataset, Poulson and colleagues determined the cytotoxicities of long-chain bases (sphingosine, dihydrosphingosine, and phytosphingosine) for human oral gingival epithelial (GE) keratinocytes, oral gingival fibroblasts (GF), dendritic cells (DC), and squamous cell carcinoma (SCC) cell lines [1]. Poulson and colleagues found that GE keratinocytes were more resistant to long-chain bases as compared to GF, DC, and SCC cell lines [1]. In this study, we assess the susceptibility of DC to lower concentrations of long chain bases. 0.2–10.0 µM long-chain bases and GML were not cytotoxic to DC; 40.0–80.0 µM long-chain bases, but not GML, were cytotoxic for DC; and 80.0 µM long-chain bases were cytotoxic to DC and induced cellular damage and death in less than 20 mins. Overall, the LD_50_ of long-chain bases for GE keratinocytes, GF, and DC were considerably higher than their minimal inhibitory concentrations for oral pathogens, a finding important to pursuing their future potential in treating periodontal and oral infections.

Specifications table

**Table:** 

Subject area	*Biology.*

More specific subject area	*Oral biology and innate immunity*.
Type of data	*One figure containing immunohistochemical micrographs and two figures containing graphs*.
How data was acquired	*Microscope and spectrophotometer (SpectraMax M2e Multi-Mode Microplate Reader, Molecular Devices, LLC, Sunnyvale, CA)*.
Data format	*Analyzed data*.
Experimental factors	*Sphingosine, dihydrosphingosine, and phytosphingosine were diluted from* 0.2–80.0 µM *for cytotoxicity assays in dendritic cells.*
Experimental features	0.2–10.0 µM *long-chain bases and GML were not cytotoxic to DC;* 40.0–80.0 µM *long-chain bases, but not GML, were cytotoxic for DC; and* 80.0 µM *long-chain bases were cytotoxic to DC and induced cellular damage and death in less than* 20 mins.
Data source location	*College of Dentistry, The University of Iowa, Iowa City, IA 52242, USA.*
Data accessibility	*There is no data in public repository.*

**Value of the data**

• Long-chain bases sphingosine and dihydrosphingosine are in saliva and have antimicrobial activity against oral pathogens.
• We determined the toxicity of sphingosine, dihydrosphingosine, and phytosphingosine for GE keratinocytes, GF, DC, and SCC cells.
• The LD_50_ of long-chain bases for GE keratinocytes, GF, SCC cells, and DC were considerably higher than their MIC for oral pathogens.
• This finding is important in pursuing the future potential of long-chain bases in treating periodontal and oral infections.

## 1 Data, experimental design, materials and methods

### 1.1 Solutions, media, and long-chain bases

0.01 M sodium phosphate with 0.14 M NaCl, pH 7.2 (PBS) was used as a diluent and as a control solution. Serum-free Lymphocyte Growth Medium 3 (LGM-3, Lonza Walkersville, Inc., Walkersville, MD) was used to cultivate GE keratinocytes, GF, DC, and SCC cells. Sphingosine (d-sphingosine), dihydrosphingosine (d-erythro-dihydrosphingosine), and phytosphingosine were prepared as recently described [1] and diluted in PBS to 640.0 μM stock solutions.

### 1.2 Cell culture

Primary, first passage gingival epithelial (GE) keratinocyte cell lines (GE363, GE367, GE368, GE369, GE370, and GE371) and primary first passage oral fibroblast cell lines (GF365, GF367, GF368, and GF369) prepared in a previous study [2] were used in this study [1]. The identity of these cells was verified by immunohistochemistry.

1.0×10^5^ viable cell/ml primary human myeloid DC (StemCell Technologies, Inc., Vancouver, BC Canada) were purchased and used in this study [1] and University of Michigan squamous cell carcinoma (SCC) cell lines SSC-15, SCC-19, SCC-84, SCC-99, and SCC-1483 [3] were also used in this study [1].

### 1.3 Histopathologic analysis

Immunohistochemistry (IHC) was performed to confirm the identity of isolated keratinocytes and fibroblasts. For this, GE keratinocytes and GF were fixed in 10.0% neutral buffered formalin overnight at room temperature; pelleted by centrifugation at 400*g* (Eppendorf 5810R centrifuge, Brinkmann Instruments, Inc., Westbury, NY); suspended in molten 2.0% BactoAgar-2.5% gelatin suspension as described [4]; and routinely processed for microtomy. Blocks were dehydrated in 70, 80, 95, and 100% ethanol solutions; clarified (Pro-Par Clearant™, Anatech, Ltd., Battle Creek, MI); embedded in paraffin; sectioned at 4 μm; and stained with HE.

IHC was performed by the University of Iowa Diagnostic Laboratories (University of Iowa, Iowa City, IA) using antibodies to vimentin (Dako, Carpenteria, CA; dilution 1:900) and pancytokeratin (Dako, Carpenteria, CA). The pancytokeratin cocktail contained antibodies to AE1/AE3 (dilution 1:200), cytokeratin 7 (dilution 1:100), and cytokeratin 8/18 (dilution 1:200).

### 1.4 Cytotoxicity assays

The effects of long-chain bases on cell metabolism (conversion of resazurin to resorufin), membrane permeability (uptake of propidium iodide or SYTOX-Green), release of cellular contents (LDH) were all determined as described [1].

To assess the effects of long-chain bases on DC cell metabolism in culture, 200 µl LGM-3 containing 2.0×10^4^ viable cells were put into each well of a 96-well microtiter plate and allowed to attach at 37 °C in 5% CO_2_. After 2 h, the cell culture media and nonadherent cells were removed, and LGM-3 with resazurin (Alamar Blue, Invitrogen Corp., Carlsbad, CA) containing 10.0–640.0 µM (final concentration) long-chain base or GML was added. LGM-3 with resazurin was added to cells and served as live cell controls (LC), and LGM-3 with resazurin containing 1.0% sodium azide was added to cells and served as killed cell controls (KC). The plates were incubated at 37 °C with 5% CO_2_ for 48 h. The metabolic reduction of resazurin to resorufin was determined using an excitation wavelength of 544 nm and an emission wavelength of 590 nm (SpectraMax M2e Multi-Mode Microplate Reader, Molecular Devices, LLC, Sunnyvale, CA).

To assess the effects of long-chain bases on DC membrane permeability of cells in suspension, 200 µl LGM-3 containing 2.0×10^4^ viable cells were put into 1.5 ml polypropylene microcentrifuge tubes; gently pelleted by centrifugation at 400*g* (Eppendorf 5810R centrifuge, Brinkmann Instruments, Inc., Westbury, NY) for 10 mins at 24°C; and the supernatants were removed. Pelleted cells were suspended in LGM-3 containing 0.3–80.0 µM long-chain bases or GML. LGM-3 was added to cells and served as LC controls and LGM-3 containing 1.0 % sodium azide was added to cells and served as KC controls. The tubes were incubated at 37 °C with 5% CO_2_. At 16 h, the cells were gently pelleted by centrifugation and the supernatants were removed. 100 µl of LGM-3 plus 10 µl of propidium iodide (0.5 mg/ml, Sigma-Aldrich, St. Louis, MO) were then added to the cell pellet and gently mixed. A 10 µl aliquot of cell suspension was removed and placed in a hemocytometer for the determination of total cell count and viable cell count by fluorescence microscopy.

Lactate dehydrogenase (LDH) was determined in DC culture supernatants using the CytoTox 96 Non-radioactive Cytotoxicity Assay (Promega Corp., Madison, WI) and read at 490 nm in the spectrophotometer (SpectraMax M2e Multi-Mode Microplate Reader, Molecular Devices, LLC, Sunnyvale, CA).

### 1.5 Statistical analysis

Percent cytotoxicity was defined as the median fluorescence intensity (MFI) of resazurin in cell culture media of cells treated with dilutions of long-chain bases/MFI of resazurin in cell culture media of untreated cells×100. The lethal dose 50 (LD_50_) was determined from the dose response curve where the 50 percent cytotoxicity intercepts with the long-chain base concentration on the *x*-axis. A log10-transformation was applied to the long-chain base LD_50_ concentrations for GE keratinocytes, GF, DC, and SCC cells. The log transformation attenuates the positive skew in the distributions of the LD_50_ concentrations and makes the normality assumption more defensible. For analysis, the long-chain base LD_50_ concentrations were first transformed by adding 1 uM to each LD_50_ value. In those instances where the LD_50_ was >640.0 μM the value used for the log10-transformation was 641.0 μM. One-way fixed-effects ANOVA models were then fit to the log-transformed concentrations. The factor levels consisted of the dilution levels for each long-chain base at 48 h. Pairwise group comparisons were conducted using the method of Tukey’s Honest Significant Differences. A 0.05 level was used to determine statistically significant differences. All analyzes were conducted using JMP (Version 10.0, SAS, Cary, NC).

## 2 Data

### 2.1 Histopathologic analysis

**Fig. 1:**
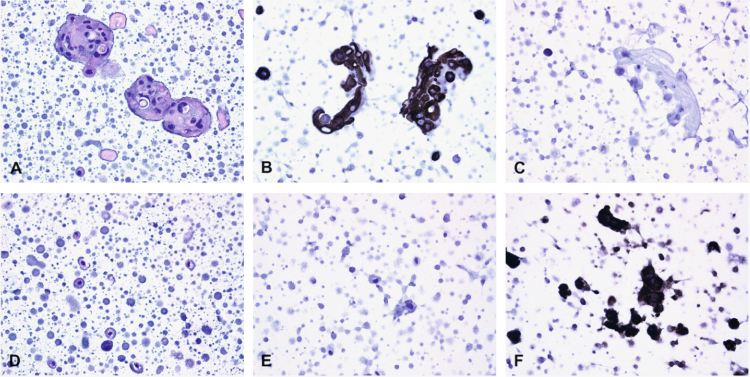
Hematoxylin and eosin (HE) staining and immunohistochemistry (IHC) were performed to confirm the identity of isolated GE keratinocytes and fibroblasts. GE keratinocyte preparations (a) stained with HE retained their cellular integrity, (b) expressed AE1/AE3, cytokeratin 7, and/or cytokeratin 8/18, and (c) did not express vimentin confirming that they were keratinocytes. GF preparations (d) stained with HE retained their cellular integrity, (e) did not express AE1/AE3, cytokeratin 7, and/or cytokeratin 8/18, and (f) expressed vimentin confirming that that these cells were of mesenchymal origin. Original magnification of 40× for all cells.

All GE keratinocytes (e.g., GE363, GE368, GE369, GE370, and GE371) expressed AE1/AE3, cytokeratin 7, and/or cytokeratin 8/18 and did not express vimentin confirming that these cells were keratinocytes (Fig. 1a–c). Likewise, GF368 and GF369 preparations did not express AE1/AE3, cytokeratin 7, and/or cytokeratin 8/18 yet did express vimentin confirming that these cells were of mesenchymal origin (Fig. 1d–f).

### 2.2 Cell cytotoxicity

The effects of long-chain bases on cell metabolism (conversion of resazurin to resorufin) were first determined and GE keratinocytes were more resistant to the three long-chain bases while GF and SCC were more susceptible (see Figs. 1 and 2 in [1] and Table 1 in [1]). GF were susceptible to the cytotoxicity of long-chain bases (see Fig. 3 in [1] and Table 1 in [1]). SCC cells, used as a control, were the most susceptible to the cytotoxicity of long-chain bases (see Fig. 4 in [1] and Table 1 in [1]).

**Fig. 2:**
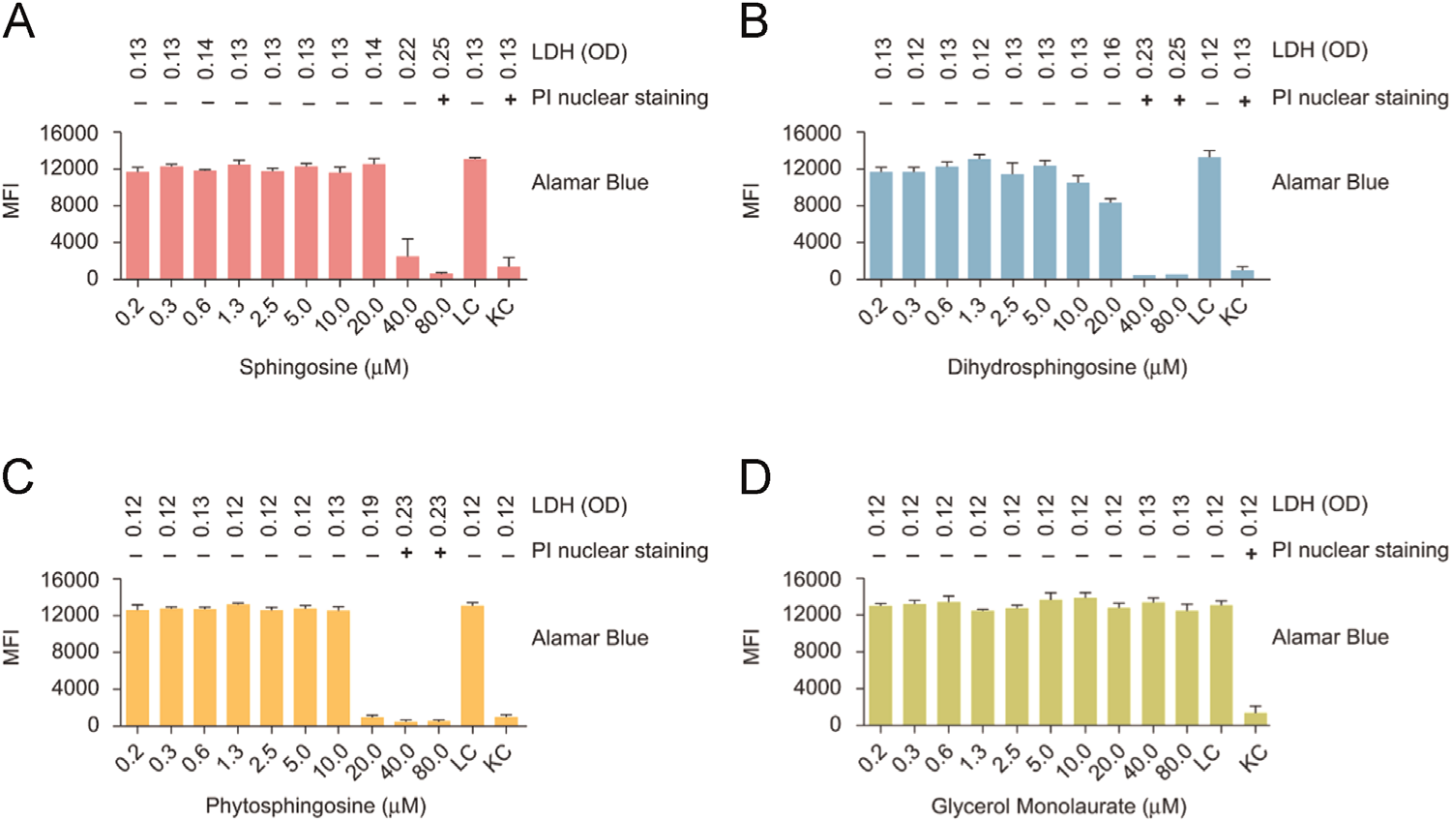
Long-chain bases and glycerol monolaurate affect the cell metabolism (conversion of resazurin to resorufin), alter membrane permeability (propidium iodide, PI nuclear staining), and release of cellular contents (lactate dehydrogenase, LDH) of a primary human dendritic cell (DC) culture. These graphs show the median fluorescence intensity (MFI) of resorufin in DC cell culture after 48 h of exposure to 0.2–80.0 µM sphingosine (A), dihydrosphingosine (B), phytosphingosine (C), and glycerol monolaurate (D). LGM-3 with resazurin was added to untreated cells and served as live cell controls (LC) and LGM-3 with resazurin containing 1% sodium azide was added to cells and served as killed cell controls (KC). Individual DC cells in suspension exposed to 0.2–80.0 µM sphingosine (A), dihydrosphingosine (B), phytosphingosine (C), and glycerol monolaurate (D) were also stained with propidium iodide and supernatants of DC cells in suspension were also checked for LDH content.

DC were also susceptible to long-chain bases (see Fig. 5, in [1]). However, there was heterogeneity among the cell lines in their response to sphingosine (cytotoxicity range 16.8–287.1 μM; mean LD_50_ 32.8 μM±3.1 μM std err), dihydrosphingosine (cytotoxicity range 13.9–58.9 μM; mean LD_50_ 24.5 μM±1.3 μM std err), phytosphingosine (cytotoxicity range 8.1–>40.0 μM; mean LD_50_ 15.2 μM±0.3 μM std err), and GML (cytotoxicity range 108.8–>160.0 μM; mean LD_50_ >80.0 µM (Fig. 2). Therefore the cytotoxicity of physiologic (5.0 µM) and high (80.0 µM) concentrations of sphingosine, dihydrosphingosine, phytosphingosine, and GML were studied in depth at 0 to 60 mins with flow cytometry and confocal microscopy.

**Fig. 3:**
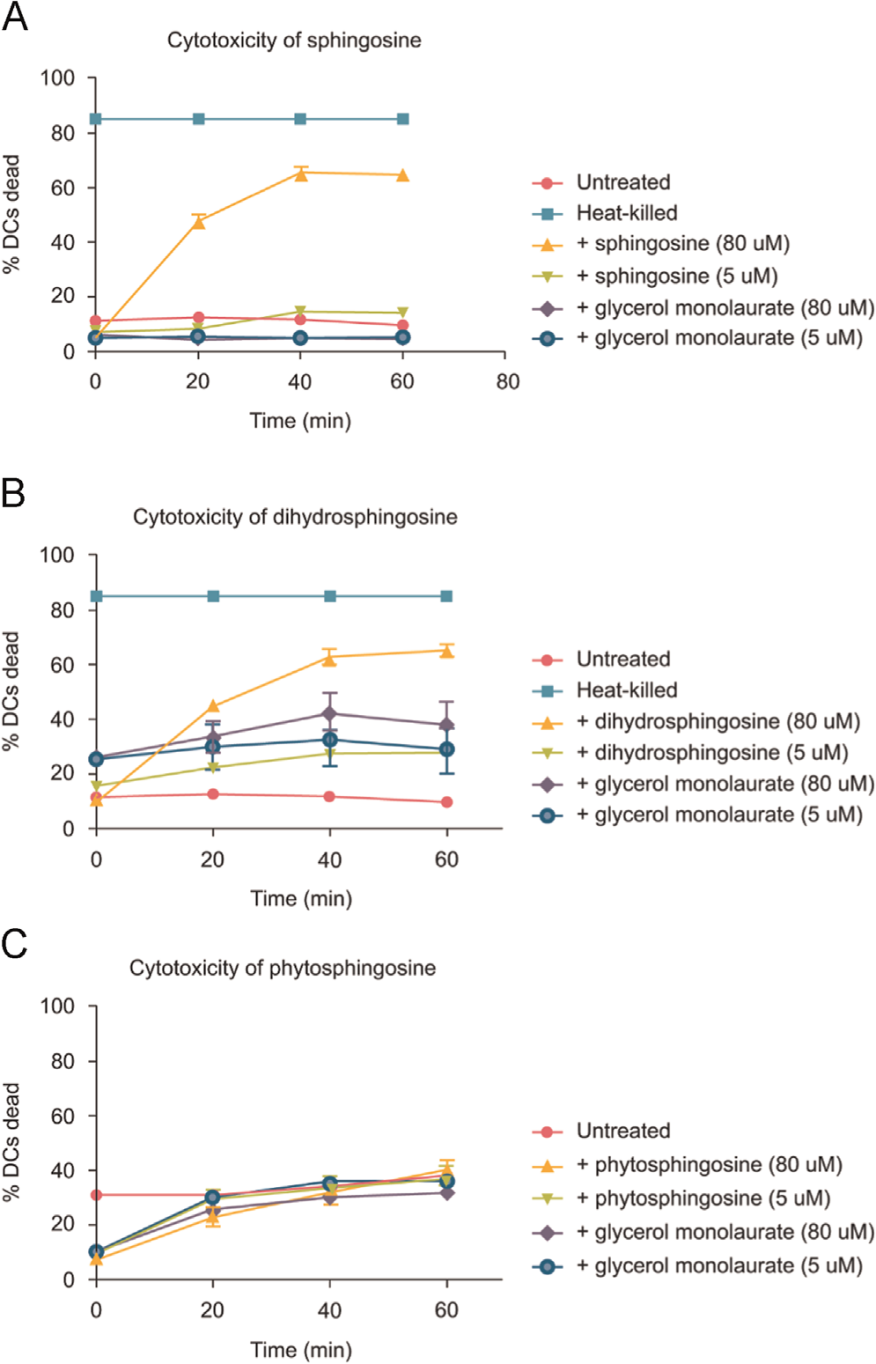
Long-chain base and glycerol monolaurate-induced cytotoxicity was assessed with flow cytometry. 600 µl LGM-3 containing 1.4–2.4×10^5^ viable DC/ml (in suspension) were first treated with 500 nM C_12_-Resazurin and 10 nM SYTOX-Green (LIVE/DEAD^®^ Cell Vitality Assay Kit, Molecular Probes, Eugene, OR) and then with 5.0 or 80.0 µM long-chain base and glycerol monolaurate. At 0, 20, 40, and 60 mins, suspended cells were examined using a LSR II Flow Cytometer (BD Biosciences, San Jose, CA). Each time point represents an average of three individual longitudinal experiments. Error bars represent SEM; where no error bars are present the SEM was approximately zero.

In flow cytometry, DC treated with 5.0 and 80.0 µM long-chain bases and GML had both concentration-dependent and time-dependent shifts in the numbers of cells with SYTOX-Green stained nuclei (see Fig. 6a–h in [1] and Fig. 3). 5.0 µM sphingosine and GML were not cytotoxic and 5.0 µM dihydrosphingosine and phytosphingosine were only mildly cytotoxic whereas 80.0 µM sphingosine, dihydrosphingosine, and phytosphingosine were cytotoxic. 80.0 µM GML concentrations were not cytotoxic. The majority of DC were dead within 20–40 mins of exposure (see Fig. 6d, f, h in [1]).
